# For Sea Sickness

**Published:** 1890-10

**Authors:** 


### FOR SEA SICKNESS.

Charles W. Hamilton, a naval surgeon, publishes this plan for curing sea sickness : The successful treatment of sea sickness, which surgeons afloat have so much to do with, and which generally they are unable effectively to alleviate, must prove my excuse for bringing before the profession the curative effect of kola. (*Sterculia acuminata.*) In the few cases which I have lately had to deal with, I have found the internal administration of the seed of the kola a most successful remedy. Half to one drachm of the seed chewed slowly was followed, in about forty minutes, by complete cessation of the various symptoms of *mal de mer*, the depression, vomiting and giddiness disappeared ; the heart's action was regulated and strengthened, and a confidence was felt in heavy weather that my cases never before experienced during the many years that they served in the royal navy, and had tried the usual remedies prescribed by their advisers. At present, no means of preventing sea sickness in those susceptible of it is known ; and I venture to believe that in the kola, or its alkaloid, we have one, and that a larger trial of this drug will tend to support my opinion. From its well known sustaining and invigorating properties during fatigue, for which it is daily used by the natives on the west coast of Africa and the Soudan, its action in sea sickness seems to be the giving tone to the nervous system, proving a stimulant—acting generally and locally.

